# *Drosophila mef2* is essential for normal mushroom body and wing development

**DOI:** 10.1242/bio.035618

**Published:** 2018-08-16

**Authors:** Jill R. Crittenden, Efthimios M. C. Skoulakis, Elliott S. Goldstein, Ronald L. Davis

**Affiliations:** 1 McGovern Institute for Brain Research, Department of Brain and Cognitive Sciences, Massachusetts Institute of Technology, Cambridge, MA, 02139, USA; 2 Division of Neuroscience, Biomedical Sciences Research Centre ‘Alexander Fleming’, Vari, 16672, Greece; 3 School of Life Science, Cellular, Molecular and Bioscience Program, Arizona State University, Tempe, AZ, 85287, USA; 4 Department of Neuroscience, The Scripps Research Institute Florida, Jupiter, FL 33458, USA

**Keywords:** MEF2, Mushroom bodies, Brain, Wing, Venation, *Drosophila*

## Abstract

MEF2 (myocyte enhancer factor 2) transcription factors are found in the brain and muscle of insects and vertebrates and are essential for the differentiation of multiple cell types. We show that in the fruit fly *Drosophila*, MEF2 is essential for the formation of mushroom bodies in the embryonic brain and for the normal development of wings in the adult. In embryos mutant for *mef2*, there is a striking reduction in the number of mushroom body neurons and their axon bundles are not detectable. The onset of MEF2 expression in neurons of the mushroom bodies coincides with their formation in the embryo and, in larvae, expression is restricted to post-mitotic neurons. In flies with a *mef2* point mutation that disrupts nuclear localization, we find that MEF2 is restricted to a subset of Kenyon cells that project to the α/β, and γ axonal lobes of the mushroom bodies, but not to those forming the α’/β’ lobes.

## Abstract (precis)

**Summary:** *Drosophila mef2* expression is restricted to subsets of mushroom body neurons, from the time of their differentiation to adulthood, and is essential for mushroom body formation.

## INTRODUCTION

Gene duplications can lead to functional variations among family members, thereby driving increased cell-type diversity (Arendt, 2008) and evolutionary pressure to maintain replicates (Assis and Bachtrog, 2013). To understand the most basic functions of a gene family it is expedient to evaluate functions that are conserved across species. The MEF2 family of transcription factors has been assigned a myriad functions ranging from the differentiation of multiple cell lineages during development, to cellular stress response and neuronal plasticity in adulthood. *Drosophila* has just one *mef2* gene, compared to the family of four *mef2* genes in vertebrates, and can thus provide insight to conserved functions of this family. As in vertebrates, *mef2* in *Drosophila* is critical for the differentiation of multiple muscle cell lineages and is essential for viability (Lilly et al., 1995; Lin et al., 1997; Potthoff and Olson, 2007). However, the role of *Drosophila mef2* in neuronal development remains untested.

*Drosophila mef2* and vertebrate *mef2* members exhibit considerable diversity in their transcriptional activation domains, but over 80% identity in the N-terminal sequences that encode the dimerization and DNA binding MEF and MADS domains (named for the evolutionarily conserved founding members MCM1, AGAMOUS, DEFICIENS, SRF) (Molkentin et al., 1996; Potthoff and Olson, 2007). Correspondingly, the DNA sequences bound by MEF2 are evolutionarily conserved and MEF2 has been shown to activate transcription of orthologous gene sets in flies and mice (Bour et al., 1995; Lilly et al., 1995; Ranganayakulu et al., 1995; Lin et al., 1997; Potthoff and Olson, 2007).

In vertebrates, the tissue specificity of MEF2's actions in muscle, brain and the immune system is strongly influenced by the expression pattern of co-factors and other MEF2 family members (Desjardins and Naya, 2017). Depending on which transcription factors MEF2 interacts with, immortalized cells in culture can be induced to display variable cell phenotypes: MEF2 and myogenin activate each other's expression to initiate differentiation into skeletal muscle, MEF2 and Nkx2.5 activate each other's expression to induce cardiac muscle formation, and MEF2 and MASH1 activate each other's expression to yield a neuronal phenotype (Skerjanc et al., 1998; Ridgeway et al., 2000; Skerjanc and Wilton, 2000). In mammalian neurons, a complex array of functions have been found for *mef2* family members in both development and neuroplasticity (Mao et al., 1999; Okamoto et al., 2000, 2002; Flavell et al., 2006; Shalizi et al., 2006; Li et al., 2008; Ryan et al., 2013; Okamoto et al., 2014; Chen et al., 2016). Studies of neuronal *mef2* in a species with a single ortholog serve to simplify this complexity by elucidating *mef2*’s most conserved functions.

*Drosophila mef2* is expressed in Kenyon neurons (Schulz et al., 1996) that make up the mushroom body (MB), a brain structure known for its functions in learning and memory [for review see Busto et al. (2010) and Cognigni et al. (2017)]. Kenyon neurons arise from four neuroblasts that divide throughout embryonic, larval and pupal development (Lee et al., 1999) to form bilateral clusters of cells located in in the dorso-posterior part of the brain. Kenyon cells extend single neurites anteriorly to form the MB calyx, pedunculus, and lobes. The MB calyx is located just anterior to the Kenyon cell bodies and comprises a plexus of MB neuropil intertwined with inputs from sensory systems. The pedunculus is formed from fasciculated MB axons that extend to the anterior portion of the brain where the axons branch to form lobes that extend either medially or vertically. In adult *Drosophila*, the Kenyon neurons can be classified into three major types depending on their axonal branching pattern: the α/β type forms the vertically-extending α lobe and the medially-extending β lobe, the α’/β’ type forms the vertically-extending α’ lobe and the medially-extending β’ lobe, and the γ type forms a single medially-extending lobe (Crittenden et al., 1998; Tanaka et al., 2008). Each axonal lobe is segregated into domains according to their interconnections with distinct types of cholinergic MB output neurons and neuromodulatory dopaminergic neurons (Aso et al., 2014). Numerous genes required for olfactory learning are preferentially expressed in the MBs, often in subsets of axonal lobes that likely reflect their distinct functions (McGuire et al., 2001; Yu et al., 2006; Krashes et al., 2007; Akalal et al., 2010; DasGupta et al., 2014; Lim et al., 2018).

Here, we examine the expression of MEF2 in the developing MB and among subsets of Kenyon cells in the adult fly, and evaluate MB formation and phenotypes in *mef2* mutant alleles.

## RESULTS

### Enhancer-detector lines identify *mef2* regulatory regions

**Fig. 1.**
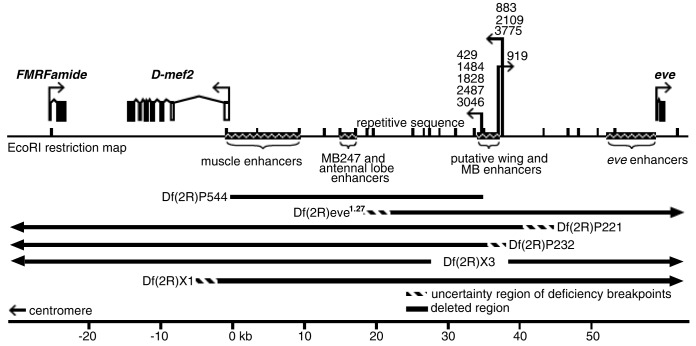
**Enhancer-detector insertion sites upstream of *Drosophila mef2*.** Insertion sites of the P element (P[lArB]) are shown for the nine enhancer-detector lines. A single arrow represents independent insertions that are within 200 base pairs of each other. The direction of the arrows indicates the direction of *lacZ* transcription, which encodes β**–**galactosidase. The locations of fragment MB247, which drives expression in MB and antennal lobe, and a fragment that drives expression in muscle, were derived from Schulz et al. (1996). The putative location of a second MB enhancer, and an enhancer for the developing wing, are defined by the expression and phenotypes we found in the enhancer-detector lines. The *mef2, FMRFamide* and *eve* gene structures and the breakpoints of the deletions are based on previous studies (Bour et al., 1995; Lilly et al., 1995; O'Brien et al., 1994; Schulz et al., 1996). Open boxes of the *mef2* transcription unit represent untranslated exons and filled boxes represent exons in the open reading frame.

From approximately 100 first-generation, P-element enhancer-detector lines (Bellen et al., 1989; Wilson et al., 1989) that were selected for β**–**galactosidase reporter activity in the MB (Han et al., 1996), we identified nine with insertions in cytological region 46C3 (Fig. 1). We mapped the insertion sites by isolating plasmid rescue clones (Pirrotta, 1986; Wilson et al., 1989) and using restriction mapping and DNA hybridization to compare to the 46C locus map (O'Brien et al., 1994; Bour et al., 1995; Lilly et al., 1995). For all nine lines, the insertions were within a 3.5 kb region that is approximately 35 kb upstream of the *mef2* transcription start site (Fig. 1). Although the insertions sites were independent, they were clustered into two regions with those closest to *mef2* showing preferential β**–**galactosidase activity in the MB and antennal lobes and those farther away showing additional expression throughout the cortex of the central brain and the optic lobes (Fig. S1).

**Fig. 2.**
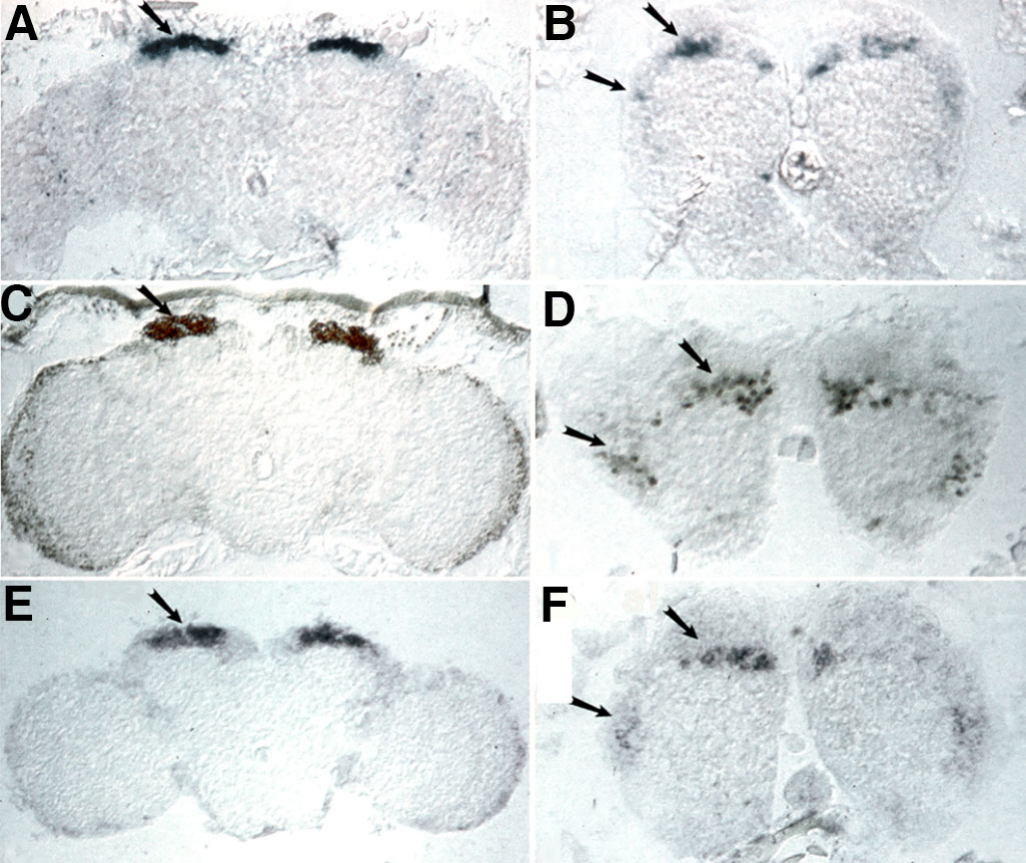
***mef2* mRNA and protein are enriched in adult mushroom body and antennal lobe neurons.** Frontal sections through the adult brain showing the corresponding localization of β–galactosidase activity, the *mef2* transcript, and MEF2 protein. Arrows in panels A,C and E designate the MB cells. Arrows in B,D and F designate cells dorsal and lateral to the antennal lobe glomeruli. (A,B) β–galactosidase activity in cryosections from line 2487. (C,D) Immunohistochemistry showing MEF2 protein distribution in paraffin-embedded sections. (E,F) RNA *in situ* hybridization on cryosections showing *mef2* transcript distribution. More than five flies were used for each experiment.

We compared the β–galactosidase expression pattern to that of *mef2* mRNA and protein in adult brain sections and found concordant enrichment in Kenyon cells and antennal lobe neurons (Fig. 2A–F). These data suggest that reporter expression in the 46C enhancer-detector lines is under the control of *mef2* MB and antennal lobe enhancers. A 2.2 kb genomic fragment that is located between the *mef2* transcription start site and the enhancer-detector elements (Fig. 1) was previously found to drive MB expression (Schulz et al., 1996), this fragment was used to generate *Drosophila* Gal4 line MB247 and other lines with various expression patterns in the MB (Schulz et al., 1996; Zars et al., 2000; Riemensperger et al., 2005; Pitman, 2011; Pech et al., 2013). However, the deficiency Df(2R)P544, which was derived from enhancer-detector line 2487 and lacks DNA sequence between *mef2* and the 2487 insertion site (Fig. 1), retained preferential β–galactosidase expression in the MB (not shown), suggesting that there are at least two MB enhancer sequences at 46C (Fig. 1).

### Characterization of *mef2* point mutants

Considering the expression of *mef2* in the adult MB and the key role for *mef2* in muscle development, we sought to investigate whether *mef2* mutants show defects in MB morphology. All nine of the *mef2* enhancer-detector lines showed grossly normal MEF2 expression and MB morphology (not shown). We therefore turned to mutants that were previously shown to disrupt *mef2* function based on lack of complementation for viability with deficiencies that encompass *mef2* (Bour et al., 1995; Goldstein et al., 2001). Nine of these lines were generated by chemical mutagenesis with ethyl methanesulfonate (*mef2^22–21^*, *mef2^22–24^*, *mef2^25-34^*, *mef2^26-6^*, *mef2^26-7^*, and *mef2^26-49^*) or diepoxybutane (*mef2^30-5^*, *mef2^44-5^*, and *mef2^48-7^*), and two were generated by γ-ray mutagenesis (*mef2^66-65^* and *mef2^78-11^*). The sites of DNA mutation were previously identified for five of the lines: *mef2^22–21^* carries a point mutation that changes the 6th amino acid position into a stop codon (Bour et al., 1995), point mutations within the MADS box domain convert Arg to Cys at amino acid position 15 in *mef2^25-34^* (Nguyen et al., 2002) and Arg to Cys at amino acid position 24 in *mef2^26-6^* and *mef2^26-7^* (Nguyen et al., 2002; Lovato et al., 2009), and *mef2^26-49^* carries a point mutation that converts Thr to Ala at position 148 (Lovato et al., 2009). To generate hypomorphic adult flies for phenotypic evaluation, we performed *inter se* complementation tests for viability (Table S1). We found that some alleles were strong (0% viability in combination), some medium (1–40% viability in any combination), and others weak (>40% viability in any combination). All of the escaper flies showed MEF2 expression and grossly normal MB morphology as adults (not shown); however these fly lines were valuable for informative experiments described below.

### MEF2 is expressed in mushroom body neurons that send axonal projections into the α/β and γ lobes

**Fig. 3.**
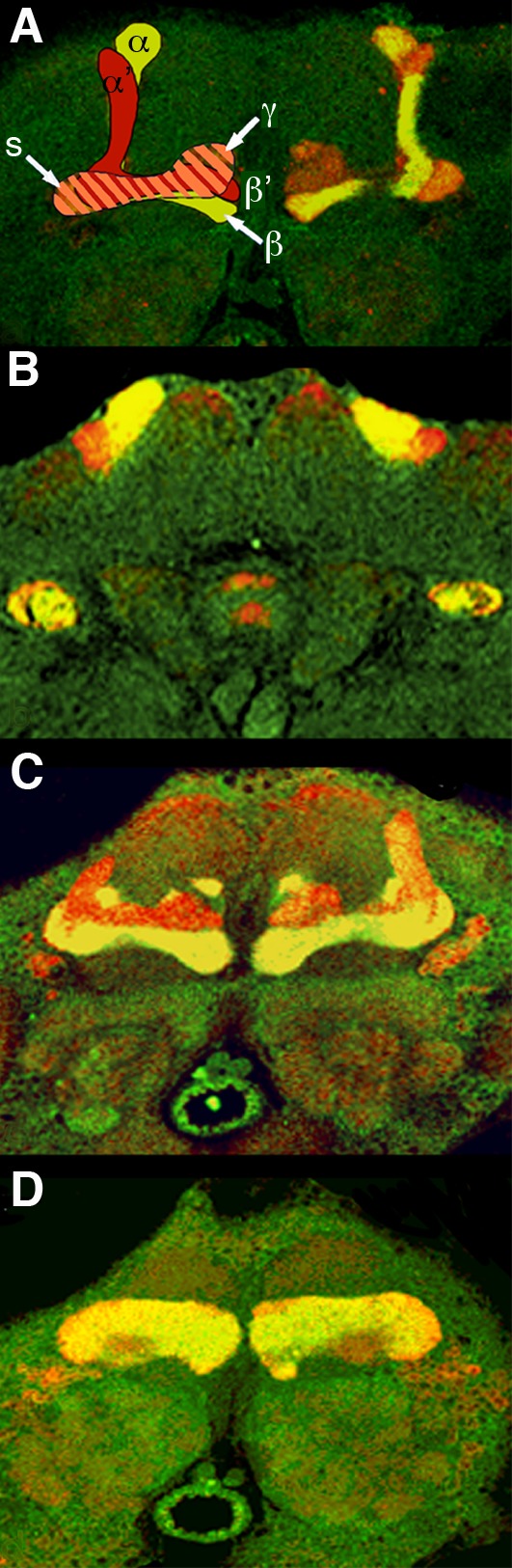
**MEF2 is expressed in mushroom body neurons that project to the α, β and γ lobes, but not the α’ and β’ lobes.** (A) A frontal paraffin-embedded section through the central brain of a wild-type animal with an illustrative drawing over the lobes on one side. MB lobes were identified by gross anatomy and immunomarkers. LEO and FASII were detected by rabbit and mouse primary antisera, respectively, which were visualized with corresponding secondary antibodies coupled to red fluorophore (for LEO) or green fluorophore (for FASII). Regions with co-expression of LEO and FASII appear yellow. LEO is present in all five lobes and FASII is in the α/β lobe branches but not the α’/β’ lobes. The spur (s) and the posterior tips of the γ lobes are defined by light FASII immunoreactivity. (B–D) In the *mef2^26-49^* line (homozygote shown here), frontal paraffin-embedded brain sections from (B) posterior to (D) anterior are co-immunolabeled for cytoplasmic MEF2 (green) and LEO (red). Co-labeling is apparent in the α/β and γ lobes (yellow) whereas the α’/β’ lobes are not co-labeled for MEF2. More than five flies were found to have a similar pattern of expression.

In our evaluation of *mef2* mutants we discovered that in line *mef2^26-49^*, MEF2 is mislocalized to the cytoplasm. In *mef2^26-49^* mutants, MEF2 immunoreactivity decorated the axons of the α/β and γ lobe-projecting neurons but was absent from the α’/β’ lobes (Fig. 3A–D). This finding is consistent with our observation in wild-type flies that several clusters of MB neurons lacked MEF2 immunoreactivity as determined by double-labeling with anti-LEONARDO (LEO), an immunomarker that exhibits global MB expression (Skoulakis and Davis, 1996).

**Fig. 4.**
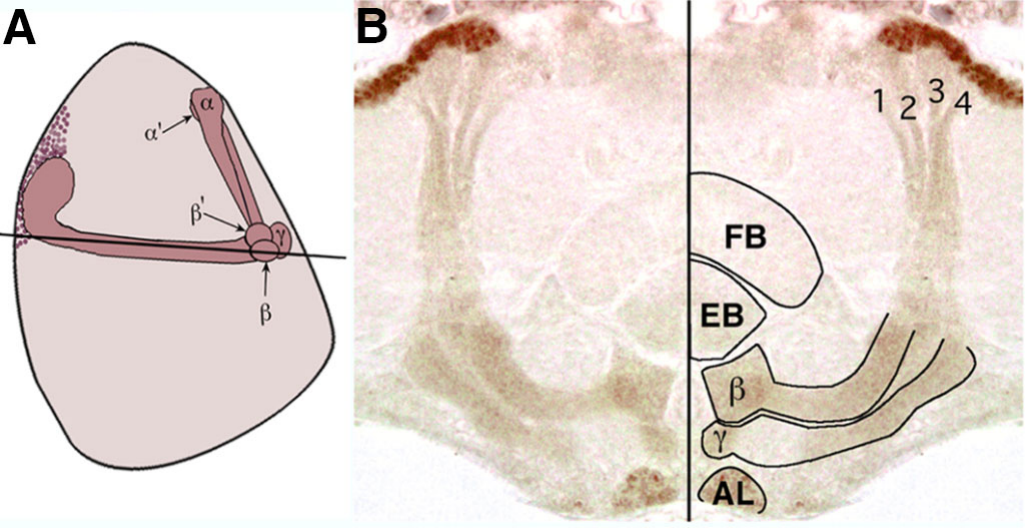
**MEF2 is expressed in Kenyon cell descendants from all four mushroom body neuroblasts.** (A) A cartoon of the adult MB in a sagittal plane, with anterior to the right. The black horizontal line represents the approximate plane of the section shown in B. (B) A near-horizontal section from a heterozygous *mef2^26-49^* adult shows immunoreactivity (brown) in all four axon bundles of the posterior pedunculus. In the mirrored image, the four axon bundles arising from the Kenyon cells are numbered and the antennal lobe (AL), fan-shaped body (FB), ellipsoid body (EB), and MB lobes (β and γ) are outlined. More than five flies were confirmed to have similar results.

In horizontal brain sections from heterozygous *mef2^26-49^* mutants, MEF2 immunoreactivity was apparent in all four bundles of the posterior pedunculus (Fig. 4A,B), each of which is formed from the progeny of a single MB neuroblast (Lee et al., 1999). Thus, *mef2* is expressed in the descendants of all four MB neuroblasts, but only those that project axons into the α/β branched lobes and into the γ lobes.

In the antennal lobe of *mef2^26-49^* flies, cytoplasmic MEF2 appeared restricted to the glomeruli and was not observed in projections of antennal lobe neurons (Fig. 4B and additional data not shown), consistent with MEF2 expression in antennal lobe interneurons. In the mutants, cytoplasmic MEF2 immunoreactivity was also detected in branches of the antennal nerve that extend into the antenno-mechanosensory center and into the antennal lobe (not shown), neurons that arise from the 2nd and 3rd antennal segments, respectively (Power, 1946). Correspondingly, nuclei within both antennal segments exhibited MEF2 immunoreactivity, a pattern also shown by the β-galactosidase expression in the enhancer-detector lines (not shown). Other cells with MEF2 immunoreactivity in the head included muscles, photoreceptor cells, most cells of the lamina, and cells distributed throughout the medulla, lobula, and lobula plate.

### MEF2 is expressed in subsets of embryonic and larval mushroom body neurons

**Fig. 5.**
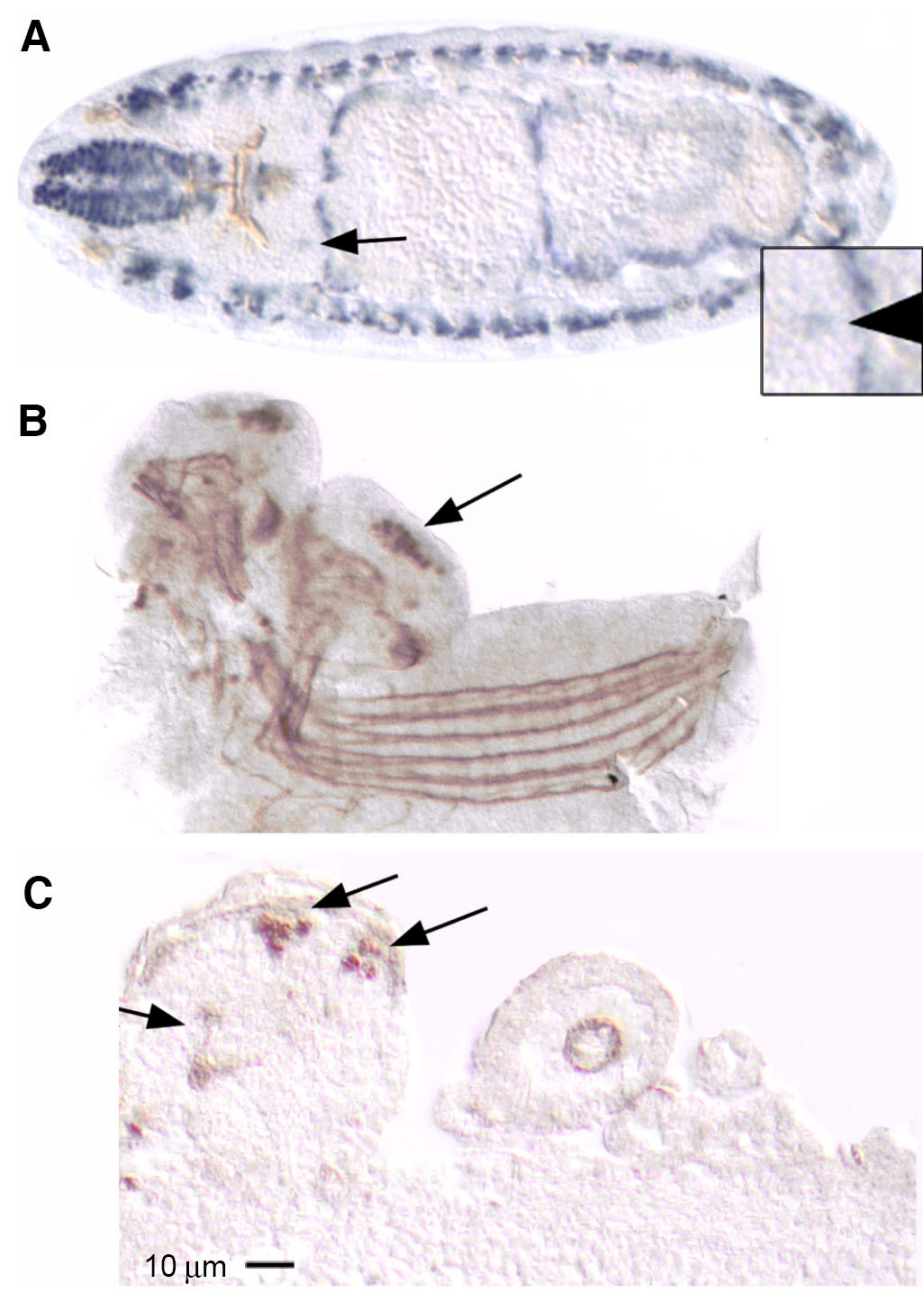
**MEF2 is expressed in embryonic mushroom bodies.** (A) A horizontal section through a stage 15 wild-type embryo embedded in plastic and immunostained for MEF2 (alkaline phosphatase-coupled secondary antibody, blue) and FASII (horseradish peroxidase-coupled secondary antibody, brown). MEF2 expression is abundant in somatic and visceral muscle cell nuclei and is also visible in bilaterally symmetrical cells of the dorso-posterior brain where MB neurons are localized (arrow and magnified in inset). FASII labels axon tracts throughout the developing nervous system whereas MEF2 is localized to cell nuclei. (B) A wholemount of the central nervous system dissected from a late stage 17 wild-type embryo and immunolabeled for MEF2 and FASII (both detected with horseradish peroxidase-coupled secondary antibody substrate, brown). MEF2 and FASII immunoreactivity is distinguished by the respective localization to nuclei and axons. MEF2 expression is highly enriched in the MB nuclei (arrow). The brain is slightly turned so that both hemispheres are equally visible. (C) A sagittal paraffin section through a late stage 17 embryo that is heterozygous for the *mef2^26-49^* mutation in which MEF2 is mislocalized to the cytoplasm. MEF2 immunoreactivity is apparent in the pedunculus and vertical lobe (arrow) and MB nuclei (arrows in dorso-posterior brain). Anterior is to the left in A–C. At least three embryos showed similar results for each experiment.

To explore the onset of *mef2* expression in the MB, we surveyed expression from early stages of development. MEF2 was detectable in one or two cells in the dorso-posterior brain at embryonic stage 15 (Fig. 5A) and the number had grown by stage 17 (Fig. 5B), which is consistent with expression in a cell type that is proliferating in late embryogenesis. Indeed, MB neuroblast proliferation is evident from stage 13 to late stages of embryogenesis (Truman and Bate, 1988; Ito and Hotta, 1992; Prokop and Technau, 1994; Kunz et al., 2012). In heterozygous *mef2^26-49^* embryos, which display cytoplasmic MEF2 immunoreactivity, there was neuropil labeling in the brain that resembled the MB pedunculus and vertical lobe (Fig. 5C). Double-labeling experiments with antibodies against MEF2 and against the Kenyon cell markers DACHSHUND (DAC) (Kurusu et al., 2000; Martini and Davis, 2005) and against EYELESS (Kurusu et al., 2000; Noveen et al., 2000; Kunz et al., 2012) showed only a partial overlap with MEF2 (not shown). We concluded that MEF2 is expressed in a subset of newly born Kenyon cells, from stage 15 to stage 17 of embryogenesis.

**Fig. 6.**
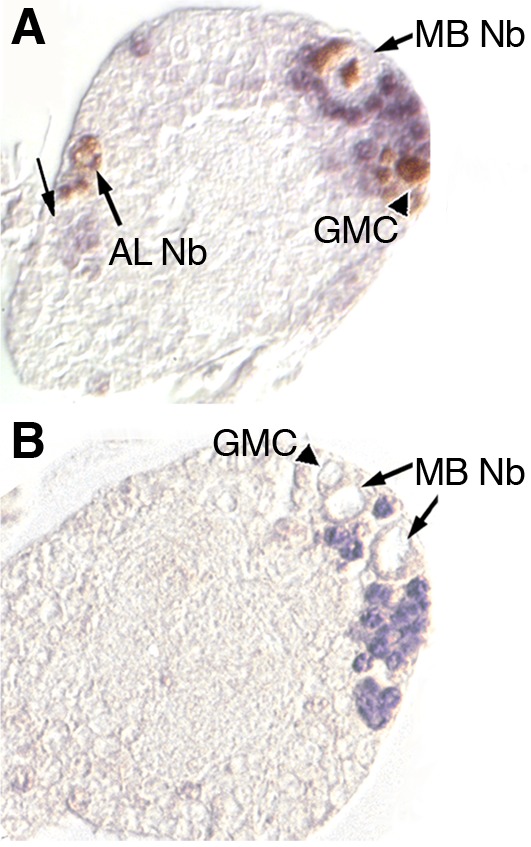
**MEF2 is expressed in mushroom body neurons, but not their neuroblast or ganglion mother cell precursors.** (A) A sagittal paraffin-embedded section through the brain of a first instar larva fed BrdU immediately after hatching and then immunolabeled for BrdU (brown) and MEF2 (blue); anterior is to the left. Anti-BrdU labels the nuclei of MB neuroblasts (MB Nb) and antennal lobe neuroblast (AL Nb) and their daughter cells, including a putative ganglion mother cell (GMC, arrowhead). Highly specific anti-MEF2 labeling is apparent in cell nuclei surrounding the MB neuroblast, and more weakly staining cell nuclei are visible near the antennal lobe neuroblast (left-most arrow). (B) A first instar larval brain section immunolabeled only for MEF2 shows the absence of MEF2 in neuroblasts and a putative ganglion mother cell. At least three larvae were evaluated for each experiment.

At the first instar larval stage, MEF2 expression was confirmed to be in the post-mitotic Kenyon cells but not in the neuroblasts or ganglion mother precursor cells (Fig. 6A,B). Weak MEF2 expression was also visible in cells surrounding, but not within, the single dividing neuroblast in the anterior brain (Fig. 6A) that is known to give rise to a variety of antennal lobe cell types (Ito and Hotta, 1992; Stocker et al., 1997; Lai et al., 2008). In short, MEF2 was found in post-mitotic Kenyon cells and antennal lobe cells, but not in neuroblasts or ganglion mother cells of the developing larval brain.

### *mef2* is required for embryonic mushroom body formation

Considering that *mef2* was expressed in the embryonic MB, we tested for MB malformation in homozygous *mef2* mutants that die as late stage embryos. We examined two different lines as embryos*,* the protein-null mutant *mef2^22-21^*, and *mef2^26-^*^6^, which carries a point mutation that disrupts the DNA binding domain but retains MEF2 expression (Nguyen et al., 2002). Although cuticle formation appeared to occur at the same time in the homozygous mutant embryos and in the heterozygous controls (with balancer chromosome), gut distension was a prominent *mef2* mutant phenotype (Ranganayakulu et al., 1995) in the homozygotes. Homozygotes were further distinguished from heterozygous controls by the absence of muscle immunolabeling for MEF2 in *mef2^22-21^* embryos and myosin heavy chain in *mef2^26-6^* embryos (Bour et al., 1995; Lilly et al., 1995).

**Fig. 7.**
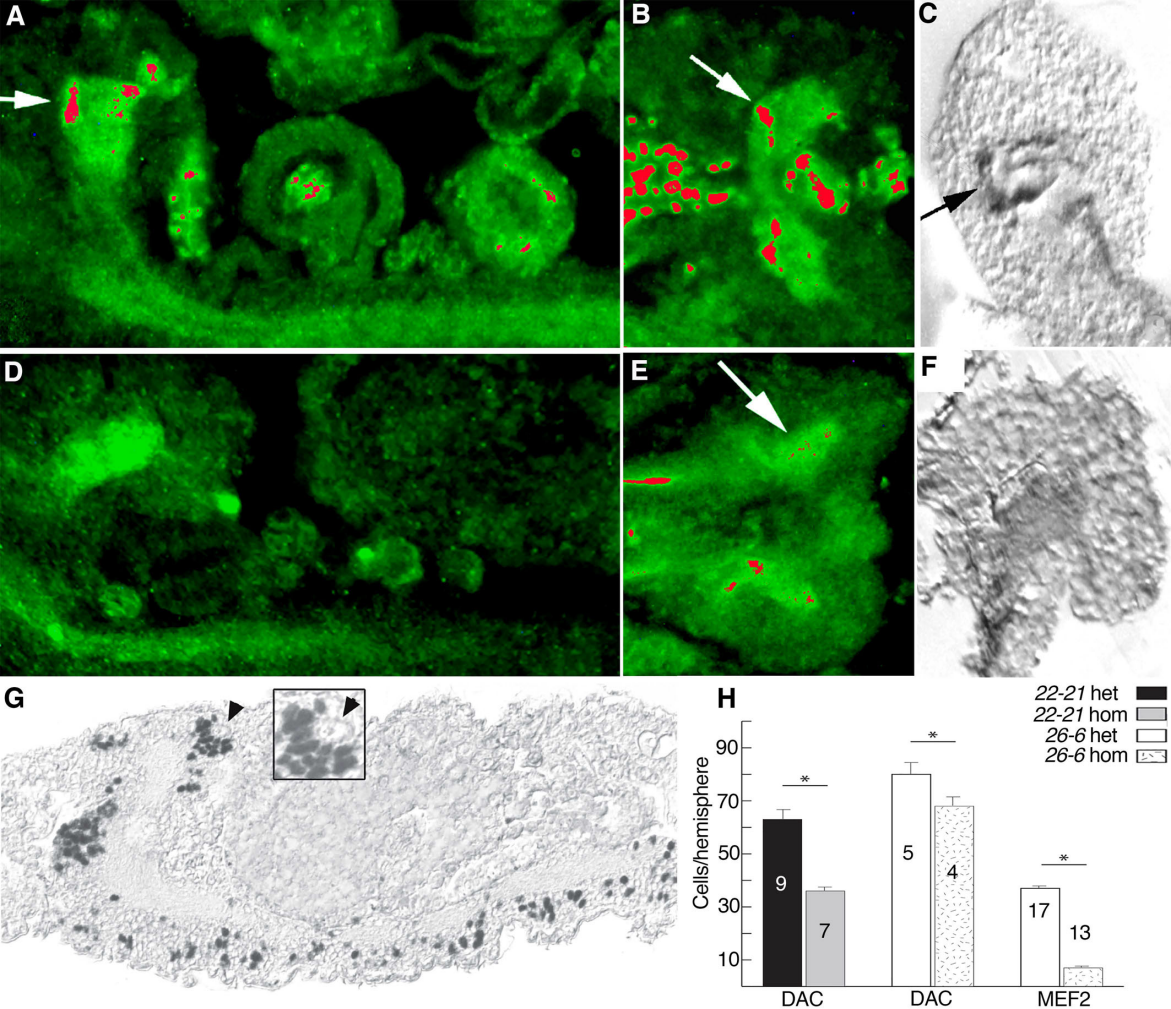
***mef2* mutant embryos have a paucity of mushroom body neurons.** Sections from stage 17 embryos that are (A–C) heterozygous balanced *mef2^22-21^* controls or (D–F) homozygous *mef2^22-21^* mutants. Section orientations are (A,D) sagittal through the entire central nervous system of paraffin-embedded tissue, (B,E) horizontal through the brain of paraffin-embedded tissue and (C,F) sagittal through the brain of plastic-embedded tissue, all with anterior to the left. (A,B,D,E) Anti-DC0 decorates the central nervous system neuropil in both genotypes (green, with highest intensity false-colored in red) but the MB lobes are visible only in controls (vertically-extending lobe at arrow in A and medially-extending lobe at arrow in B). Anti-MEF2 (also in green) labels only the cell nuclei of muscles and MB neurons in controls, not the MB axonal lobes, and was included for genotyping purposes. (C,F) Anti-FASII labels the cervical connectives the MB pedunculus, and vertical MB lobe of controls (arrow in C) but not homozygous mutants (F). (G) Example of a sagittal paraffin-embedded section through a *mef2^22-21^* embryo immunostained for DAC (black) that was used to count MB neurons located in the dorso-posterior brain (magnified in inset). The MB neuroblast is not labeled for DAC (arrowhead). (H) Counts of MB neurons that were immunolabeled for DAC or MEF2. *mef2^22-21^* and *mef2^26-6^* homozygous mutant embryos had significantly fewer MB neurons than their age-matched heterozygous balancer-chromosome controls (*P<0.05 for each pair-wise comparison by Student's unpaired two-tailed *t*-test). The number of brain hemispheres evaluated is indicated on each column. Error bars show standard errors of the mean.

We assessed MB morphology by immunolabeling with two embryonic MB markers, the protein kinase A subunit DC0, and FASII (Skoulakis et al., 1993; Crittenden et al., 1998; Cheng et al., 2001). In stage 17 heterozygous *mef2^22-21^* embryos, the immunostained pedunculus and lobes (Fig. 7A–C) appeared similar to what we showed with these and other markers previously in wild-type embryos (Crittenden et al., 1998). In contrast, neither anti-DC0 nor anti-FASII labeled MB structures in any sections from homozygous *mef2^22-21^* embryos processed on the same slides as controls (Fig. 7D–F). It is possible that the failure to see MB immunostaining in the mutants is because MEF2 regulates the expression of these markers. We tested this possibility by ectopic MEF2 expression using the *GAL4/UAS* system, with five different drivers, but did not observe ectopic expression of DC0 or FASII (not shown). These experiments are not definitive, however, because the ectopic expression might have been only in tissues that do not express a necessary co-factor for MEF2 activity. Therefore, we sought a second approach to measure MB formation.

We counted MB nuclei in *mef2^22-21^* and *mef2^26-6^* embryos. We used the MB immunomarker DAC to count MB neurons in consecutive sagittal sections from stage 16 heterozygous (with balancer) and homozygous *mef2^22-21^* animals (Fig. 7G). Anti-DAC immunoreactivity was observed in an estimated average of 63 cells per dorso-posterior brain hemisphere in the heterozygotes, compared to only 36 cells per hemisphere in the *mef2^22-21^* homozygotes (Fig. 7H), representing a 43% reduction in the number of DAC-positive MB neurons. This loss was not consequent of failed neuroblast formation, as in the process of cell counting we observed four neuroblasts in each hemisphere of the *mef2^22-21^* homozygotes. We also confirmed that these neuroblasts are dividing, based on BrdU incorporation after injection at 19 h after egg laying (not shown). We made similar cell counts in stage 16 heterozygous (with balancer) and homozygous embryos from line *mef2^26-^*^6^. The control heterozygotes had an average of 80 DAC positive MB neurons, whereas the homozygous mutants had an average of 68 (Fig. 7H), representing a 15% reduction. We also counted the number of MEF2-positive neurons in *mef2^26-^*^6^ embryos. An average of 37 cells were counted per dorso-posterior hemisphere in the controls, whereas only seven were found on average in the homozygous mutants (Fig. 7H), an 81% reduction. The difference in the number of DAC positive cells between the *mef2^22-21^* and *mef2^26-6^* heterozygous animals is likely to be due to a slight difference in the ages of the animals between experiments; however, since the heterozygous and homozygous animals within each genotype were aged and collected together, our primary evidence that there are fewer MB neurons in homozygous *mef2^22-21^* and *mef2^26-6^* mutants was not compromised.

In summary, severe hypomorphic or protein-null *mef2* mutants have reduced numbers of differentiated MB neurons based on immunolabeling with four MB markers (DAC, MEF2, FASII and DC0).

### *mef2* is required for normal wing development

**Fig. 8.**
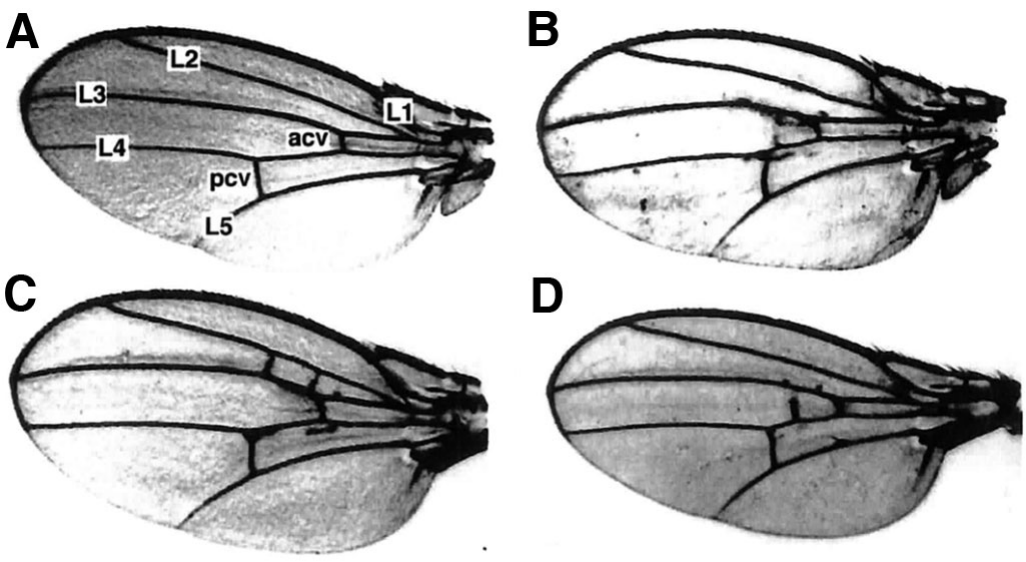
***mef2* is required for normal wing venation.** (A) A wild-type wing with veins labeled. Acv, anterior cross-vein; pcv, posterior cross-vein; L1-L5, longitudinal veins. (B) Ectopic venation and broadened wing shape in a transheterozygous *mef2^26-49/78-11^* fly. (C) Ectopic venation in homozygous enhancer-detector line 919. (D) Non-complementation of the wing-phenotype in a transheterozygous *mef2^22-21^*/line 919 fly.

In adult escapers with point mutations in *mef2* (Table S1) we often observed disrupted wing morphology ranging from incomplete or ectopic cross-veins to bubbled wings (Fig. 8A,B). Furthermore, enhancer-detector line 919 showed strong expression and complete penetrance of wing venation defects (Fig. 8C). A similar phenotype, at lower penetrance and expressivity, was observed in the enhancer-detector lines with insertions clustered more proximally to *mef2* (lines 429, 919, 1484, 1828, 2487, 3046) but not in lines with insertions more distal to *mef2* (lines 883, 2109, 3775). To confirm that *mef2* dysfunction is responsible for the wing phenotype in the enhancer-detector lines, we performed complementation tests with the protein-null mutant *mef2^22-21^*. We observed wing blistering or abnormal venation in 74% of the transheterozygotes with line 919 (Fig. 8D) and in 58% of transheterozygotes with line 429. Heterozygotes for the enhancer-detector insertions or *mef2^22-21^* did not show a wing phenotype. Our results suggest that there is an enhancer for *mef2* expression in the developing wing that spans the P element insertion site in line 919 and extends proximally toward *mef2* (Fig. 1), and establish a role for *mef2* in wing development.

## DISCUSSION

### Nuclear retention signal for MEF2

Mammalian MEF2 contains several sequences near the C-terminus that are required for its nuclear localization, but these sequences are not conserved in *Drosophila* and the MEF2 nuclear localization sequence has not been identified (Yu, 1996; Borghi et al., 2001). We identified a mutant, *mef2^26-49^*, in which MEF2 fails to be retained in the nucleus. The mutation in line *mef2^26-49^* was previously described as a missense point mutation that converts amino acid 148 from Thr to Ala (Lovato et al., 2009). From a BLAST^®^ comparison to mouse MEF2 it appeared that this Thr is conserved in MEF2A but not in other MEF2 family members. This region of the protein is evolutionarily conserved and is termed the HJURP-C domain (Holliday junction regulator protein family C-terminal repeat). The HJURP-C domain is present in MEF2A, MEF2C and MEF2D but is lacking in MEF2B. The function of the HJURP-C domain is poorly understood but our results suggest that it contributes to nuclear localization of MEF2.

### Mushroom body expression pattern of *mef2*

Previous reports have shown that MB neurons begin to differentiate at stage 14 and continue to be born until shortly before pupal eclosion (Ito and Hotta, 1992). Our embryonic expression studies indicated that MEF2 becomes detectable in the MB neurons as early as stage 15. In the embryo and larva, MEF2 immunoreactivity was in post-mitotic Kenyon cells and antennal lobe neurons, but not in neuroblasts or ganglion mother cells, consistent with the developmental expression profile of MEF2 in the honeybee *Apis mellifera* (Farris et al., 1999). Likewise, in mammals the initiation of *mef2* expression in cortical neurons coincides with their exit from the cell cycle (Lyons et al., 1995; Mao et al., 1999). Thus, the expression profile of *Drosophila* and mammalian *mef2* is consistent with a role in neuronal cell identity or differentiation.

MB neurons that give rise to the different lobes are generated sequentially from the four dorsal posterior neuroblasts and are interdependent for pathfinding and survival (Kurusu et al., 2002; Martini and Davis, 2005). In adults, we found MEF2 expression in all four tracts of the posterior pedunculus, indicating MEF2 expression in descendants of all four MB neuroblasts. Based on double-labeling experiments with other Kenyon cell markers, MEF2 is expressed in only a subset of MB neurons in the embryonic and adult stages. The cytoplasmic mislocalization of MEF2 in line *mef2^26-49^* served to show that MEF2 is expressed in Kenyon cells that form medially- and vertically-extending lobes in the embryo. In the adult, MEF2 is expressed in α/β- and γ-lobe forming neurons, but not in the α’/β’ neurons. Accordingly, the *Drosophila* Gal4 line MB247, which uses a 2.2 kb fragment upstream of *mef2* (Fig. 1) to control Gal4 expression, preferentially drives expression in the α/β and γ lobes, but not the α’/β’ lobes; however, other transgenic fly lines with this *mef2* fragment drive more widespread MB expression (Schulz et al., 1996; Zars et al., 2000; Riemensperger et al., 2005; Pitman, 2011; Pech et al., 2013). Thus, *Drosophila mef2* appears to harbor multiple MB enhancers (Fig. 1), and possibly suppressors for α’/β’ MB cell type expression as well.

Mutant cytoplasmic MEF2 showed that the antennal lobe expression appeared to be confined to interneurons whereas projection neurons were found in the antennal segments that house olfactory receptors, hygroreceptors, thermoreceptors and the sound-sensing Johnston's organ (Stocker, 1994). These MEF2-expressing structures are serially linked in the pathway for odor perception (Power, 1946): odor detection occurs in olfactory neurons of the third antennal segment, which synapse onto projection neurons in the antennal lobe glomeruli that in turn send sensory information to the MB calyces. Thus, MEF2 might function in the transmission and integration of olfactory information to, and within, the MB.

MEF2 interacts physically with myogenic and neurogenic factors to potentiate cell-type specific gene transcription (Molkentin et al., 1995; Black et al., 1996; Mao and Nadal-Ginard, 1996). The MEF2 MB lobe expression pattern expression gives clues to possible transcriptional interactors for MEF2. Examples of MB markers with similar Kenyon cell subtype distribution to MEF2 include FOXP (DasGupta et al., 2014), HDAC4 (Fitzsimons et al., 2013), DRK (Crittenden et al., 1998; Kotoula et al., 2017), and FASII (Crittenden et al., 1998; Cheng et al., 2001). MEF2 interactions with several of these molecules have already been established. In mammals, HDAC4 (histone deacetylase 4) is known to bind to MEF2 to repress transcription, and *Drosophila* HDAC4 is important for muscle development, circadian rhythmicity and MB function (Zhao et al., 2005; Fogg et al., 2014). A shared function for MEF2 and FASII (the fly ortholog of NCAM) in cell-cell communication or adhesion is suggested by our finding that *mef2* hypomorphs exhibit an ectopic venation phenotype similar to that reported for *fasII* loss of function mutant cell clones (Mao and Freeman, 2009). Furthermore, MEF2 regulates *fasII* expression in clock neurons to control their circadian fasciculation and defasciculation for the regulation of motor output (Blanchard et al., 2010; Sivachenko et al., 2013). A function for MEF2 in neuronal defasciculation raises a possible parallel to MEF2's role in synapse elimination in cultured mouse neurons (Flavell et al., 2006). FOXP proteins (forkhead box transcription factors) are also known to function in synapse elimination. Mammalian FOXP2 co-localizes with MEF2C early in development but subsequently suppresses MEF2C expression in the striatum (Chen et al., 2016), a dopamine rich forebrain region that is important for motor learning and that has compartmental organization (Crittenden and Graybiel, 2017) that has been directly compared to the MB (Strausfeld and Hirth, 2013). Overall, these studies are consistent with distinct cellular functions for MEF2 in development, and later in learning. Disruption of FOXP in the α/β MB neurons results in motor problems and delayed decision-making in an associative olfactory-discrimination task (DasGupta et al., 2014; Lawton et al., 2014) but whether this involves MEF2 remains untested.

### *mef2* function in mushroom body formation

Deletion of murine *mef2* family members impairs normal development of neurons, lymphocytes, bone, endothelial cells, and photoreceptor cells (Mao et al., 1999; Potthoff and Olson, 2007; Andzelm et al., 2015; Latchney et al., 2015). We have now shown that *mef2* is essential for the development of MB neurons. Loss of *mef2* led to a failure in MB formation, and a reduction in MB neuron number, in all of the homozygous *mef2* mutant embryos that we examined. We could not detect any MB neuropil in the *mef2* protein-null embryos with the immunomarkers anti-DC0 and anti-FASII, indicating either that the remaining DAC-positive Kenyon cells failed to extend processes or that they were too sparse to detect. Modifiers of the phenotype are suggested by the fact that escaper transheterozygous flies showed grossly normal MB morphology as adults. FASII mutations were found to disrupt MB development in one study but not in another (Cheng et al., 2001; Kurusu et al., 2002), further highlighting such phenotypic variability in MB development. It is also possible that *mef2* is important for the development of embryonic MB but not adult MB, in parallel to the finding that *mef2* serves a broader function in the formation of embryonic muscles than in adult muscles (Baker et al., 2005).

In the homozygous line *mef2^26-6^*, there was a 15% reduction in DAC-positive MB cells and an 81% reduction of MEF2-positive MB neurons. One possibility for the reduced number of MB neurons labeled for MEF2, relative to DAC, is that the *mef2^26-6^* mutation disrupts MEF2 expression. We and others (Nguyen et al., 2002) observed similar levels of MEF2 immunoreactivity in the remaining cell nuclei of *mef2^26-6^* homozygous embryos, but it is still possible that a subset of cells fail to express the mutant isoform to detectable levels. Another explanation for the severe loss of MEF2-positive MB neurons in *mef2^26-6^* homozygous embryos is that this subtype of MB neuron is more severely impacted. It was previously shown that DAC is expressed in only a subset of embryonic MB neurons (Kunz et al., 2012) and we found, by double-immunolabeling for DAC and MEF2 in controls, that some MB neurons express DAC and not MEF2 (not shown). We did not determine whether all MEF2-positive neurons express DAC. In short, the MB markers that we used are not universally expressed among embryonic MB neurons and so if the loss of MEF2 differentially impacts one subtype, differences in the proportions lost based on counts with each marker would be expected.

We considered three possible explanations for the reduced MB cell number in *mef2* mutants. First, the MB neurons may die prematurely. Second, the MB neuroblasts may fail to proliferate normally. Third, the neurons may not differentiate properly, owing either to a fate change or to a block in the differentiation program. To test whether the primary cause of reduced MB cell numbers in *mef2* mutants was cell death, we employed the vital dye Acridine Orange. Acridine Orange was applied to homozygous *mef2^26-6^* animals at stages of 14, 15, and 16, periods preceding and including the time at which mutants exhibited a clear reduction in the number of MB neurons. At stage 14, a tight cluster of cells in the dorso-posterior brain was stained with Acridine Orange in both heterozygous and homozygous *mef2^26-6^* animals. By stage 15 and 16 this staining had subsided however, leaving fewer labeled cells that were scattered throughout the CNS (not shown). Although we observed Acridine Orange staining in the muscle cells of *mef2^26-6^* homozygous embryos as previously reported (Ranganayakulu et al., 1995), we did not detect an increase in cell death within the brains of the mutants compared with controls. Therefore, we did not find evidence of abnormally increased apoptotic cell death in the MB neurons of *mef2* mutants. Nor was the *mef2* MB phenotype caused by the failure of neuroblasts to form: all four MB neuroblasts were apparent at stage 17 in *mef2^22-21^* animals as determined by counting experiments. Moreover, the neuroblasts did not express *mef2* and did incorporate BrdU, although we cannot rule out that BrdU incorporation was slowed. In conclusion, we propose that the reduction in the number of MB neurons in *mef2* mutants may best be explained by a failure of these cells to form or differentiate properly, which is consistent with their failure to form MB lobes.

### *mef2* functions in wing venation

The enhancer-detector lines led to our discovery of a wing venation function for *mef2*. The 46C enhancer-detector lines did not show gross myogenesis or MB development problems but did show ectopic wing venation and wing bubbling that is non-complementary with *mef2* point mutations and that appears identical to what we found in transheterozygous *mef2* point mutant escapers. Overexpression of *mef2* was found in a large-scale screen of transcription factors, to induce wing blistering (Schertel et al., 2015) but it was not investigated further. Screens for wing venation phenotypes have identified over 300 genes with enrichment for members of the Notch, EGFR and Dpp (TGF-β homolog) signaling pathways that are critical for intercellular communication (Molnar et al., 2006; Bilousov et al., 2014). MEF2 can be linked to the regulation of these pathways. For example, *Tkv* (thick veins), which encodes a Dpp receptor, is repressed by MEF2 during *Drosophila* egg formation (Mantrova et al., 1999). Indeed, disruptions in *Dpp* and *Tkv* expression can result in anterior cross-vein and blistering phenotypes (de Celis, 1997) that are similar to what we observed in *mef2* hypomorphs. Another member of the Dpp-Tkv pathway is p38 mitogen-activated protein kinase, which can phosphorylate and activate mammalian MEF2 (Han et al., 1997; Mao et al., 1999; Okamoto et al., 2000) and in its dominant-negative form causes ectopic wing venation in flies (Adachi-Yamada et al., 1999). Collectively with our results, these data suggest that the abnormal vein formation in hypomorphic *mef2* mutants is caused by a failure in the Dpp-Tkv pathway.

## MATERIALS AND METHODS

### *Drosophila* genetics

Fly stocks were raised at room temperature on standard sucrose and cornmeal media. The nine enhancer-detector lines described were identified in a screen for MB expression (Bellen et al., 1989; Wilson et al., 1989; Han et al., 1996). Both male and female adult flies were used and embryos were not sexed. The EMS, DEB, and γ-ray mutants shown in Table S1 were identified in a screen for lethal genes at the cytological location 46C–F (Goldstein et al., 2001). The parental chromosome for these lines was *adh cn pr* and they were maintained balanced over *CyO*. The *CyO* mutation impacts wing formation so for complementation analysis of adult viability and the wing phenotype, the lines in Table S1 were rebalanced with the homozygous lethal chromosome SM6BevelacZ that has a dominant rough-eye marker (Roi). The lack of a rough-eye phenotype was used to identify transheterozygous *mef2* mutants.

### Molecular biology

Bacteriophage clones surrounding the enhancer-detector insertion site in line 2487 were isolated from a Canton-S genomic library. The map constructed of the 46C region was expanded by 12 kb from coordinate 20 kb to 32 kb (Fig. 1) relative to the previously published maps (Bour et al., 1995; Lilly et al., 1995). The expansion was due to a stretch of repetitive DNA suggesting the likely insertion of a transposable element. Genomic DNA fragments adjacent to the insertions in lines 429, 883, 919, 2487, 3046, and 3775 were obtained by *Hind III* or *XhoI* plasmid rescue, according to previously described methods (Pirrotta, 1986). The insertion sites in lines 1484, 1828, and 2109 were determined by Southern blotting experiments.

### Histology

β**–**galactosidase histochemistry and RNA *in situ* hybridization experiments were performed on frontal cryosections of the *Drosophila* head as previously described (Skoulakis and Davis, 1996). For comparative evaluation of β**–**galactosidase activity, multiple flies from each line were examined and reacted for similar amounts of time. RNA probes were generated from the 5′ and the 3′ end of a *mef2* cDNA and used in separate experiments to validate RNA *in situ* hybridization results.

Antisera for MEF2, provided by Dr E. Olson, were raised against a fusion protein comprising amino acids 1-472 that contained both the MADS box and MEF domain of MEF2. Antibodies, with working dilutions given in parentheses, were generated in rabbit for MEF2 (1:1000) and DC0 (1:400), in mouse for FASII (1:2) and DAC (1:30), and in rat for BrdU (1:30, Harlan Sera-Laboratory). Specificity of the antibodies were previously validated by reduced immunoreactivity in flies with the corresponding mutations for *mef2* (Lilly et al., 1995), *DC0* (Lane and Kalderon, 1993; Skoulakis et al., 1993), *fasII* (Lin and Goodman, 1994; Cheng et al., 2001), and *dac* (Martini et al., 2000). Immunohistochemistry with chromogenic substrates was performed on paraffin-embedded sections from larvae and adults, or prior to plastic embedding and sectioning of embryonic *Drosophila* as previously described (Crittenden et al., 1998). For immunofluorescence, CY3- or FITC-conjugated anti-rabbit and anti-mouse antibodies (1:400, Sigma-Aldrich) were used. Slides were coverslipped with Vectashield (Vector Laboratories, Burlingame, USA).

### Cell counting experiments

Immunolabeled MB cells were apparent in approximately 15×1 µm serial sections of each brain hemisphere. Each cell was visible in an average of 3.5 serial sections. Therefore, to estimate the number of MB cells per brain hemisphere, we divided the total number of cells counted by 3.5. Statistical comparisons between homozygous and heterozygous, balancer-chromosome control embryos were made using unpaired, two-tailed Student's *t*-tests. Comparisons were always made between mutant and control embryos obtained from the same matings and processed together. No embryos were excluded from analyses after cell counting.

### Cell death assay

Acridine Orange staining was performed as previously described (Abrams et al., 1993). Homozygous *mef2* mutants were distinguished from sibling controls based on the presence of bloated gut morphology.

### BrdU labeling

The treatment of larvae with BrdU to label dividing cells followed the protocol of Truman and Bate (1988). For immunohistochemical detection of BrdU, paraffin sections of larvae were additionally treated with 2N HCl.

## Supplementary Material

Supplementary information
