# Supplementary material for: Drosophila mef2 is essential for normal mushroom body and wing development
Source: Biol Open. 2018 Aug 16;7(9):bio035618. doi: 10.1242/bio.035618 (PMC6176937; doi:10.1242/bio.035618)
Supplement: Supplementary information [file biolopen-7-035618-s1.pdf]

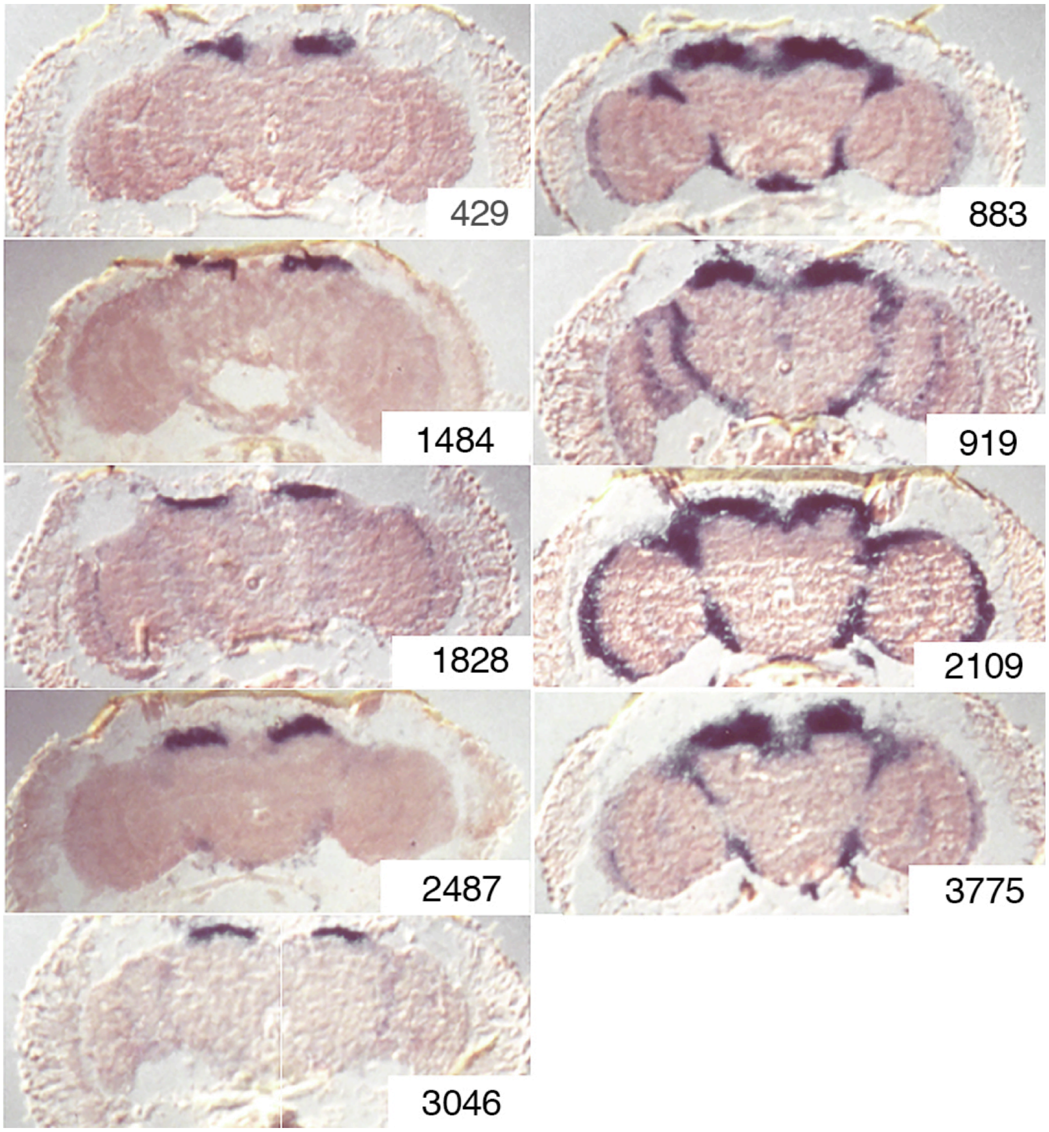

Supplementary Figure 1. Enhancer detector lines reflect mushroom body and antennal lobe expression. Frontal sections of adult fly brains showing β–galactosidase activity in the mushroom bodies of nine enhancer-detector lines with *lacZ* reporter gene insertions near *Drosophila mef2* (insertion locations shown in Fig. 1). Those lines with insertions closer to *mef2* (left column) exhibit β–galactosidase activity restricted to the mushroom body neurons whereas the lines with slightly more distal insertions (right column) exhibit additional activity throughout the cortex.

Biology Open • Supplementary information

Supplementary Table 1. Intragenic complementation for lethality in *mef2* alleles.[1]

***mef2* allele**	**22-21**	**22-24**	**25-34**	**26-6**	**26-7**	**26-49**	**30-5**	**44-5**	**48-7**	**66-65**
**78-11**	0 (192)	0 (232)	0 (217)	0 (206)	Not tested	1 (203)	13 (97)	0 (193)	0 (220)	0 (185)
**66-65**	0 (221)	0 (272)	0 (260)	0 (224)	0 (191)	0 (210)	35 (194)	0 (240)	0 (266)	
**48-7**	0 (315)	0 (307)	0 (282)	0 (230)	0 (210)	0 (203)	0 (208)	0 (304)		
**44-5**	0 (198)	1 (251)	6 (188)	0 (246)	0 (203)	28 (307)	14 (212)			
**30-5**	0 (210)	3 (358)	28 (207)	0 (121)	0 (128)	78 (131)				
**26-49**	0 (174)	0 (227)	45 (252)	0 (235)	0 (234)					
**26-7**	0 (178)	0 (406)	0 (195)	0 (195)						
**26-6**	0 (193)	0 (291)	0 (272)							
**25-34**	0 (223)	0 (598)								
**22-24**	0 (519)									

[1] Each cell shows the percentage of transheterozygous adult progeny observed out of the number expected. The total numbers of progeny recovered are indicated in parentheses.
